# Do investigator meetings improve recruitment rates in clinical trials? A retrospective before-and-after study of data from nine multi-centre clinical trials

**DOI:** 10.1186/s13063-020-04465-1

**Published:** 2020-06-10

**Authors:** E. J. Mitchell, P. J. Godolphin, G. Meakin, K. Sprange

**Affiliations:** 1 Nottingham Clinical Trials Unit, University of Nottingham, University Park, Nottingham, UK; 2 MRC Clinical Trials Unit at University College London, Institute of Clinical Trials and Methodology, London, UK

**Keywords:** Recruitment, Investigator meeting, Clinical trial

## Abstract

**Background:**

Poor recruitment in clinical trials is well-documented. In large, multi-centre trials, communication between the coordinating centre and trial sites is essential. A commonly used communication tool is the hosting of an investigator/collaborator meeting, which offers an opportunity for sites to re-train and receive trial updates, learn from each other, share best practice and troubleshoot issues. Anecdotally, there is a perception that recruitment rates may increase after holding such a meeting. The aim of this before-and-after study was to examine any changes in recruitment after an investigator meeting.

**Methods:**

We conducted a retrospective study of nine trials at the Nottingham Clinical Trials Unit (NCTU) that were open to recruitment between 2014 and 2018. In the 8 weeks prior to the date of the investigator meeting, 82 sites (across nine trials) were open to recruitment; 60 of which attended the meeting, 22 who did not. Using meeting attendance data available in Trial Master Files (TMF) and recruitment data from randomisation datasets, we examined recruitment rates in the 8 weeks prior to and following the date of the investigator meeting.

**Results:**

For the 82 sites included, 284 participants were recruited in the 8 weeks prior to the meeting, with a further 300 participants recruited in the 8 weeks post meeting. This gives a mean change in weekly recruitment of 0.073 (− 0.129, 0.275) per site, demonstrating no statistically significant increase in recruitment after the investigator meeting. For the 60 attending sites, recruitment increased from 254 participants prior to the meeting to 271 post meeting, giving a 0.100 (− 0.160, 0.360) mean change in weekly recruitment per site, providing no evidence that recruitment rates increase following an investigator meeting.

**Conclusion:**

There is no statistical evidence to conclude that holding an investigator meeting increases recruitment in the 8 weeks following the meeting. Thus, if the meeting has been held in the belief that it will have a positive impact upon recruitment, trialists may wish to consider other evidence-based strategies known to increase recruitment rates. However, since there are a variety of reasons why an investigator meeting may be held, trialists should continue to consider this as a communication strategy with sites.

## Background

Clinical trials often fail to recruit to time and on target, and issues with poor recruitment are well-documented [1]. To run a successful trial that adequately answers the research question, a large collaborative team is required, particularly for definitive trials. It is important to ensure continued communication with trial sites throughout and, although there has been no formal evaluation, it is thought that good communication with sites could have an effect upon trial recruitment [2, 3]. Groups such as the Hubs for Trials Methodology Research (HTMR) recommend maintaining engagement with clinical trial sites and holding meetings as a strategy that may improve recruitment [4]. It is commonplace for trials to hold meetings with site staff, such as the principal investigator and research nurse, who are responsible for recruitment of participants and data collection. These are often known as ‘investigator’ or ‘collaborator’ meetings. Indeed, some Clinical Trials Units (CTUs) include the costs for such meetings in funding applications and these meetings form part of their standard practices. Such meetings can be held during the recruitment and data-collection phase of a trial, and are often repeated intermittently for the duration of a trial. In some circumstances, the meetings may be held in response to trial-specific issues, such as poor recruitment. They offer an opportunity for sites to re-train, network and be part of the trial community and to share good practice or troubleshoot issues with other sites and the coordinating centre. At least anecdotally, there appears to be a belief amongst trialists that holding an investigator meeting may lead to an increase in recruitment. However, after searching a range of resources, including the Online Resource for Recruitment Research in Clinical Trials (ORRCA) [5], Trial Forge [6] and the Study Within a Trial (SWAT) repository store on the Northern Ireland Hub for Trials Methodology Research [7], along with literature searches, we found no published evidence to support this. The aim of this study was to examine whether holding an investigator meeting in multi-centre trials, managed by the Nottingham Clinical Trials Unit (NCTU), had an impact on trial recruitment.

## Methods

Using data available in Trial Master Files (TMFs) and trial recruitment data, we conducted a retrospective review of multi-centre trials from NCTU’s portfolio of trials over a 5-year period between January 2014 and December 2018. Trials that met the following criteria were included in data collection and analysis: (1) more than one site, i.e. multi-centre, (2) in the recruitment phase during the selected time period, (3) held at least one face-to-face investigator meeting during the recruitment period and (4) unit of randomisation was individual participants (any trials that included group randomisation were not included). During the selected time period, NCTU had two group-based therapy trials where groups of participants were randomised, rather than individuals. It was agreed these should not be included since recruitment issues and strategies may vary substantially from trials where individual participants are recruited. We defined an investigator meeting as a meeting where all participating United Kingdom (UK) sites were invited to attend to hear trial updates, share information and discuss issues, in accordance with the standard operating procedure at the NCTU. Trials that held smaller, local or regional meetings or only held meetings for a different purpose (i.e. to discuss study results) were not included. A simple data-collection tool using JISC online surveys^©^ was designed to collect basic details relating to any investigator meetings held during the recruitment phase of the trial. This included the characteristics of each trial, when the meeting(s) were held, which sites were in attendance and which type of site staff (i.e. job role) attended the meeting(s). The data-collection tool was user-tested by the authors and circulated to trial management staff for completion. Recruitment data for each site for the 8 weeks pre and post investigator meeting were taken directly from the randomisation datasets for each trial. Although somewhat arbitrary, this time period was selected to ensure that the maximum amount of recruitment data could be included from the studies involved and seemed an appropriate length of time that a potential effect on recruitment levels may be seen. We felt that if an effect was seen on recruitment > 8 weeks after the investigator meeting, it would be less plausible to suggest an association between the two.

For trials that held more than one meeting during the recruitment period, we used data from the meeting that was held closest to the middle of the recruitment period. The rationale for this was that meetings held towards the middle of the recruitment period should involve the largest number of sites open to recruitment, rather than meetings held towards the start of the period that may involve only a small number of actively recruiting sites or towards the end of a trial when sites may have closed to recruitment or reached their recruitment target.

### Statistical analysis

Continuous variables were summarised with mean and standard deviation (SD), or median and interquartile range (IQR). Categorical variables were described with frequency counts and percentages. Linear mixed-effects models were fitted with 16 weeks of recruitment data per site to estimate the change in recruitment after an investigator meeting with the following data structure: weeks nested within sites, with sites nested within trials. One model was fitted for all eligible sites, and a second only for sites who attended their investigator meeting. In both models, random intercepts were fitted at both trial and site level. The assumptions of the models were checked to ensure that the residuals were normally distributed, the variance was homogenous and that there was a linear relationship between the residuals and predictor. All analyses were performed in Stata version 16.0 or later.

## Results

**Fig. 1:**
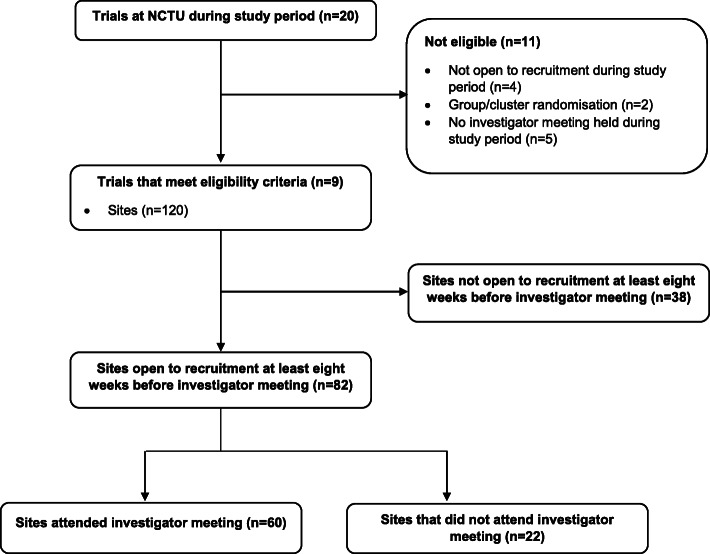
Flow diagram showing included and excluded trials

During the 5-year selected time period, there were a total of 20 trials managed by NCTU. Eleven of these did not meet our pre-specified eligibility criteria, with four not open to recruitment during the study period (4/11, 36%), five that did not hold an investigator meeting during the study period (5/11, 45%) and two that used group randomisation (2/11, 18%). Thus, nine trials were included in this study; all of which have been published or have published protocols [8–16]. One trial, the Leucopatch trial, included a small number of international sites (*n* = 10) though for consistency purposes it was felt better to only include data relating to UK sites. In total the trials comprised a total of 120 UK sites, however, 38 of these were not open to recruitment at least 8 weeks prior to the investigator meeting and, thus, these sites were not included. Eighty-two sites were open to recruitment at least 8 weeks before the investigator meeting, of which 60 attended the meeting and 22 did not (Fig. 1).

Characteristics of the included trials are shown in Table 1. The nine trials included a range of Clinical Trials of Investigational Medicinal Products (CTIMPs), device trials and complex intervention trials. Six trials were the definitive main trial and three were feasibility trials. Most (8/9, 88.9%) were funded by the National Institute for Health Research (NIHR). Trials spanned several clinical areas including dermatology (3/9), pregnancy and childbirth (2/9), diabetes (1/9), gastroenterology (1/9), hand surgery (1/9) and sexual health (1/9). Six trials recruited adults only, two were paediatric and one recruited both adults and children. The mean sample size was 549.6 participants (SD 648.6, range 50–1900) and involved, on average, 14 sites (SD 8.1, range 3–25). The mean length of recruitment time was 23.6 months (SD 11.7, range 10–44.4).

**Table 1:** Characteristics of included trials

**Characteristic**	**Trials (*n*** **= 9) (%)**
**Type of trial**	
CTIMP	3 (33%)
Device	1 (11%)
Intervention	5 (56%)
**Stage of trial**	
Definitive	6 (67%)
Feasibility	3 (33%)
**Funding type**	
NIHR	8 (89%)
Industry	1 (11%)
**Clinical area**	
Dermatology	3 (33%)
Diabetes	1 (11%)
Gastroenterology	1 (11%)
Hand surgery	1 (11%)
Pregnancy and childbirth	2 (22%)
Sexual health	1 (11%)
**Population**	
Adults	6 (67%)
Paediatric	2 (22%)
Adults and paediatric	1 (11%)
**Sample size**	
Mean (SD)	549.6 (648.6)
Median (IQR)	269 (180, 517)
Min, Max	50, 1900
**Length of recruitment (months)**	
Mean (SD)	23.6 (11.7)
Median (IQR)	22.4 (17.1, 26.1)
Min, Max	10.0, 44.4
**Number of sites**^**a**^	
Mean (SD)	13.9 (8.1)
Median (IQR)	16 (8, 22)
Min, Max	3, 25

All data are *N* (%) unless otherwise stated

*CTIMP* Clinical Trial of an Investigational Medicinal Product, *IQR* interquartile range, *NIHR* National Institute for Health Research (any programme)

^a^One trial was international and for this study only the UK sites are included

Investigator meetings were conducted throughout the calendar year, spanning all seasons. On average, there were eight sites open to recruitment for at least 8 weeks prior to the investigator meeting per trial (median 8, IQR 8–12) with seven who actually attended the meeting (median 7, IQR 7–8). For the 60 sites who attended the investigator meeting for their trial, on average two members of site staff were in attendance. It was more common for research nurses/midwives (*n* = 62, 84%) and principal investigators (36, 49%) to attend, though other roles also in attendance included co-investigators, biomedical scientists, research assistants and microbiologists; this varied for each trial.

The effect of an investigator meeting upon 8-weekly recruitment is given in Table 2. Irrespective of attendance at the investigator meeting, for the 82 sites from the nine trials included, a total of 284 participants were recruited in the 8 weeks prior to the investigator meeting being held, with 300 participants recruited in the 8 weeks following the meeting. The mean change in weekly recruitment per site was 0.073 (95% confidence interval (CI) − 0.129, 0.275). This translates to recruitment of one extra participant per 14 sites per week.

**Table 2:** Effect of investigator meeting on weekly recruitment rate 8 weeks pre and post meeting – results from linear mixed-effects models

**Population**	**Time point**	**8-weekly recruitment**	**Mean change in weekly recruitment per site (95% CI)**	***P*value**^**a**^
All sites (*n* = 82)	Pre meeting	284	0.073 (− 0.129, 0.275)	0.48
	Post meeting	300		
Sites who attended (*n* = 60)	Pre meeting	254	0.100 (− 0.160, 0.360)	0.45
	Post meeting	271		

*CI* confidence interval

^a^*p* values are from a Wald chi-square test with one degree of freedom

**Fig. 2:**
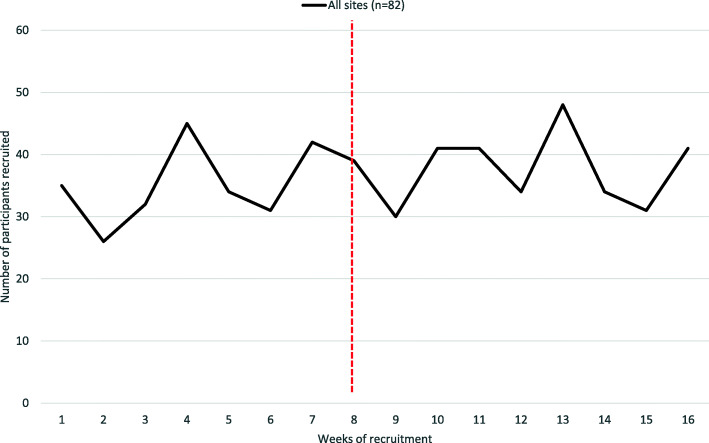
Change in recruitment rates pre and post investigator meeting (all sites)

**Fig. 3:**
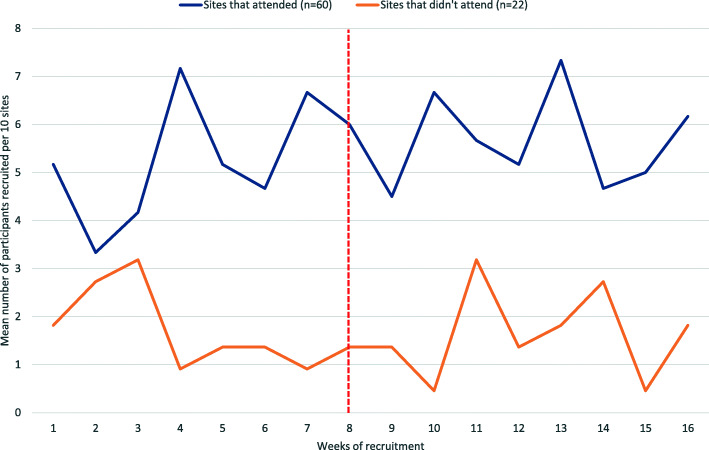
Change in recruitment rates pre and post investigator meeting (attending and non-attending sites)

For the 60 sites who attended the investigator meeting, a total of 254 participants were recruited in the 8 weeks prior to the day of the meeting, with 271 participants recruited in the 8 weeks after the investigator meeting. The mean change in weekly recruitment per site was 0.100 (95% CI − 0.160, 0.360), translating into the recruitment of one extra participant per 10 sites per week. For the 22 sites who did not attend the investigator meeting, a total of 30 participants were recruited in the 8 weeks prior to the meeting and 29 in the 8 weeks after the meeting, giving a mean change in weekly recruitment per site of 0.00 (95% CI − 0.236, 0.236). The change in recruitment rates before and after the investigator meeting, for all sites, are shown in Fig. 2 and the differences between attending and non-attending sites are shown in Fig. 3.

## Discussion

In this small retrospective study, there was no evidence to suggest that holding an investigator meeting may increase recruitment in the 8 weeks following the meeting. Although recruitment of one extra participant per week per 14 sites is a modest increase, the variability around this estimate was high such that this finding may have been due to chance.

Although the aim of this study was to investigate whether holding an investigator meeting improved recruitment rates overall, we did undertake additional exploratory analyses to examine any differences between sites who were able to attend the meeting and those who were not. Our data suggests that for sites who attend the meeting, there is a small non-significant increase in recruitment in comparison to non-attending sites, where there is no increase in recruitment after the meeting. Unsurprisingly, the sites who did not attend the meeting had lower recruitment rates, before and after the investigator meeting, than the sites who attended. This could suggest that the sites who attended the meeting had a higher level of engagement in the trial and that if recruitment is a primary concern, an investigator meeting might not be effective in reaching the target audience. It is, however, impossible to know if this was the case retrospectively.

Although there is no evidence to suggest a statistically significant increase in recruitment after an investigator meeting, nor does this data suggest a decrease in recruitment. As previously described, investigator meetings are often held for a variety of reasons and, thus, we are not suggesting that since this data supports no evidence of an increase in recruitment rates, you should not consider holding one. For example, you may wish to hold an investigator meeting for sites to learn from each other and share expertise and, therefore, funding and resource permitted, it seems sensible to continue to do this. Whilst no formal qualitative analyses have been undertaken using feedback and evaluation forms from the meetings held, all authors recognised that, anecdotally, site staff often consider attending an investigator meeting to be useful and they enjoy the opportunity to engage with other sites and the team running the trial. Investigators and trial management staff should, however, give careful consideration to holding an investigator meeting purely for the purpose of increasing recruitment.

To the best of our knowledge, this is the first time that data has been analysed to examine the effect of holding an investigator meeting on recruitment rates. This study, therefore, adds to the evidence base for strategies to improve recruitment into clinical trials. Since holding investigator meetings is common across many trials, trialists may want to consider whether their resources be targeted at evidence-based strategies shown to improve recruitment rates.

We recognise this study has some limitations. Firstly, our sample size was small with only nine trials included during a 5-year period, resulting in just 584 randomisations recorded during a 16-week period. Thus, it is possible that a larger study would detect a small but meaningful recruitment increase in the 8 weeks following an investigator meeting. Further, a larger study could explore subgroups of interest, such as time since site initiation with a hypothesis that newly opened sites may not recruit as quickly as those previously opened, and contain sufficient numbers of sites to provide a direct statistical comparison between the recruitment rates for sites who did and did not attend investigator meetings. Dependent upon the trials included in a subsequent larger study, consideration may need to be given to other factors, such as those with a longer screening period, since this may have a delayed impact upon recruitment rates which would not be attributable to attendance at an investigator meeting. Secondly, investigator meetings can be held for a variety of reasons and we have not attempted to explore the potential effects of holding a meeting on other parameters, such as data collection and staff involvement and empowerment, simply due to the data being unavailable in this retrospective study. Finally, factors that could influence site recruitment, such as site engagement and prior research experience, were not explored, since this data was not available retrospectively, though we recognise that a site’s performance is multi-faceted [17] and could influence recruitment rates.

## Conclusion

We found no statistical evidence in our study to conclude that holding an investigator meeting increases recruitment rates in the 8 weeks after the date of the meeting. Trialists should give careful consideration to the purpose and use of such meetings in the future. However, more research could be undertaken to further explore this. Ideally this would be done prospectively, though there would be challenges in implementing attendance at an investigator meeting as a SWAT intervention, for example, which may not be seen positively by trial sites. Nonetheless this study could be expanded further retrospectively by (1) considering a larger sample of trials and (2) investigating other outcomes that may be affected by holding an investigator meeting.

## Data Availability

The datasets used and analysed during the current study are available from the corresponding author upon reasonable request.

## Abbreviations

CTIMPs: Clinical Trials of Investigational Medicinal Products
CTU: Clinical Trials Unit
NCTU: Nottingham Clinical Trials Unit
NIHR: National Institute for Health Research
ORRCA: Online Resource for Recruitment Research in Clinical Trials
SWAT: Study Within a Trial
TMF: Trial Master File
